# Universal Evolution of Fickian Non-Gaussian Diffusion in Two- and Three-Dimensional Glass-Forming Liquids

**DOI:** 10.3390/ijms24097871

**Published:** 2023-04-26

**Authors:** Francesco Rusciano, Raffaele Pastore, Francesco Greco

**Affiliations:** Department of Chemical, Materials and Production Engineering, University of Naples Federico II, P.le Tecchio 80, 80125 Napoli, Italy; francesco.rusciano@unina.it (F.R.); francesco.greco@unina.it (F.G.)

**Keywords:** diffusion, glass-formers, supercooled liquids, Fickian yet non-Gaussian, Brownian motion, Brownian non-Gaussian diffusion

## Abstract

Recent works show that glass-forming liquids display Fickian non-Gaussian Diffusion, with non-Gaussian displacement distributions persisting even at very long times, when linearity in the mean square displacement (Fickianity) has already been attained. Such non-Gaussian deviations temporarily exhibit distinctive exponential tails, with a decay length λ growing in time as a power-law. We herein carefully examine data from four different glass-forming systems with isotropic interactions, both in two and three dimensions, namely, three numerical models of molecular liquids and one experimentally investigated colloidal suspension. Drawing on the identification of a proper time range for reliable exponential fits, we find that a scaling law $\lambda \left(t\right)\propto t^{\alpha }$, with α≃1/3, holds for all considered systems, independently from dimensionality. We further show that, for each system, data at different temperatures/concentration can be collapsed onto a master-curve, identifying a characteristic time for the disappearance of exponential tails and the recovery of Gaussianity. We find that such characteristic time is always related through a power-law to the onset time of Fickianity. The present findings suggest that FnGD in glass-formers may be characterized by a “universal” evolution of the distribution tails, independent from system dimensionality, at least for liquids with isotropic potential.

## 1. Introduction

Diffusion is the main transport mechanism in fluid systems, across time- and length-scales spanning from the molecular to the colloidal realms. Over the last century, different types of diffusion have been recognized and classified in different categories [1,2,3]. About a decade ago, however, such an established classification was broken up by the discovery of the so-called “Fickian yet non-Gaussian Diffusion” (FnGD), firstly observed for colloidal tracers in biological fluids [4,5]. FnGD consists in the particle Mean Square Displacement (MSD) growing linearly in time (Fickian), $\langle r^{2}\left(t\right)\rangle \propto t$, coexisting with a non-Gaussian particle displacement distribution. (While the naming “Brownian yet non-Gaussian diffusion” is also commonly adopted to indicate this phenomenon, here we prefer to use the term *Fickian* rather than *Brownian*, since the latter is less appropriate for molecular systems such as those considered in this paper.) Generally, this type of diffusion is associated to some heterogeneity of the environment where particles move, such as structural, chemical, or dynamical heterogeneity.

Following its discovery in 2009 [4], FnGD has been observed over the last decade in a variety of complex fluids and soft materials, including molecular, polymeric, colloidal and biological systems [6,7,8,9,10,11,12,13,14,15,16,17,18,19]. In most of these systems, the non-Gaussian distribution function shows exponential tails, temporarily at least. Different theoretical models to capture some features of this phenomenon have also been proposed during the last few years [20,21,22,23,24,25,26,27,28].

Quite recently, this intriguing type of diffusion has also been found in simulations of molecular glass-forming liquids [29], as well as in a combined numerical and experimental study on molecular and colloidal glass-formers [30]. It is worth emphasizing here that the persistence of non-Gaussian deviations in the long-time Fickian regime (i.e., the occurrence of FnGD) was never systematically investigated before those works, even though the presence of exponentially tailed displacement distributions in the earlier subdiffusive regime was already a well-known feature of glass-forming systems [31,32].

Both the aforementioned Refs. [29,30] show that the exponential decay-length λ of the displacement distribution tails in glass-formers does not necessarily follow the ‘diffusive-like’ time-dependence $\lambda \left(t\right)\propto t^{1/2}$, commonly found in other systems with FnGD [4,8,33,34,35,36]. Going into more detail, Miotto et al. [29] claim that, in the FnGD of glass-forming liquids, λ(t) displays a *non-universal* behaviour, depending on system features such as dimensionality *d* or the inter-particle potential. In the specific case of the paradigmatic Kob–Andersen Lennard–Jones (KALJ) model, they propose that $\lambda \left(t\right)\propto t^{1/d}$, and further speculate that this dimensionality dependence can be extended to the whole class of glass-forming liquids with isotropic potential. On the other hand, in the experimental and numerical two-dimensional glass-formers studied in [30], both pertaining to the same class of isotropic potentials, a time-dependence $\lambda \left(t\right)\propto t^{1/3}$ is found, which of course does not agree with the proposal in [29].

The discrepancy among the outcomes from the bidimensional glass-formers in [29] and [30] motivated us to re-examine the data of those systems. Specifically, the analysis to be presented here draws on a proper identification of time-window where exponential tails are well-defined, in between the onset time of Fickianity $\tau_{F}$ and the time for full recovery of Gaussianity, $\tau_{G}$. These two timescales were carefully identified in [30] and found to be related through a power-law relation $\tau_{G}\propto \tau_{F}^{\gamma }$, with γ>1, implying that the FnGD time-window enlarges on approaching the glass transition.

The paper is organized as follows. In Section 2, the four glass-former systems studied in [29,30] are shortly recalled, for readers’ convenience. These four systems are: the Kob–Andersen Lennard–Jones models in two and three dimensions [37], a model of harmonic purely repulsive disks in two dimensions [38], and a quasi-two-dimensional experimental system of hard-sphere-like colloids in water [39]. All of these are well-known glass-forming model-systems. The procedures adopted for identifying the above-mentioned characteristic timescales $\tau_{F}$ and $\tau_{G}$, as introduced in [30], are also illustrated in this Section. In Section 3.1, a direct exploration of the evolution of the displacement distribution functions in the FnGD regime is presented, to properly determine the time-range where exponential tails of the distributions are clearly defined. Thereafter, by focusing on the two KALJ molecular liquids presented in [29], it is concluded that the attribution of exponential tails to very-large-time behaviour is spurious. In other words, there exists an upper-time bound to the very presence of clear-cut exponential tails of the distributions. It is then shown in Section 3.2 that, when properly limiting the analysis to times lower than this upper bound, all KALJ data, at all temperatures, follow the same scaling, $\lambda \propto t^{1/3}$, independently from system dimensionality. As a matter of fact, we demonstrate that λ(t) data for *all* the systems considered in [29,30] obey the same scaling law, and, hence, the above-indicated discrepancy between results in [29] and in [30] is reconciled.

It so appears that a common behaviour is present for all the considered glass-formers. In the conclusions (Section 4), it is suggested that this might indeed be a *universal* feature on approaching glass transition, at least for systems with isotropic interaction potentials.

## 2. Materials and Methods

### 2.1. Investigated Systems

We have considered four glass-forming systems at equilibrium conditions, including the experimental colloidal model (2HS) and the numerical molecular model (2SD) investigated in [30], as well as the Kob–Andersen Lennard–Jones numerical model both in two (2KALJ) and three-dimensions (3KALJ) investigated in [29].

2HS is a binary Brownian suspension, at closely monolayer conditions, of micron-sized hard-sphere-like beads in water, where the dynamics slow down on increasing the volume fraction ϕ [39,40,41,42].

2SD is a simple molecular liquid model, consisting of a two-dimensional binary assembly of soft disks [38], where the dynamics slow down on decreasing the temperature *T*. Disks interact through a purely repulsive harmonic potential, as their areas overlap. Molecular Dynamics (MD) simulations at different temperatures were performed in the NVT ensemble [30].

3KALJ, introduced in [37,43], is largely the most popular numerical model of molecular supercooled liquids and consists of a binary mixture of particles interacting through a truncated and shifted Lennard–Jones potential. 2KALJ is the two-dimensional variant of the standard 3KALJ. MD simulations at different temperature were performed in the NVE ensemble, after equilibrating the system in the NVT ensemble [29]

For the three numerical systems, 2SD, 3KALJ and 2KALJ, all data presented in this work refer only to the small particles in the mixtures.

All details concerning the considered systems, as well as the experimental and simulation methods can be found in [30] for 2HD and 2SD, and in [29] for 3KALJ and 2KALJ. The variety of the interaction potentials pertaining to the considered systems together with the existence of a common dynamical behaviour of those systems highlight how “chemical details” become irrelevant for FnGD, on approaching glass-transition.

### 2.2. Time-Boundaries of FnGD Regime

For all considered systems, the lower time-boundaries for FnGD, namely the onset times of Fickian diffusion (i.e., $\langle r^{2}\left(t\right)\rangle \propto t$), are estimated as in [30]. By assuming that the characteristic length for the onset of Fickian diffusion coincides with the particle diameter $\xi_{F}=\sigma$, it comes naturally that the corresponding Fickian time is $\tau_{F}=\frac{\sigma^{2}}{2dD}$, where *D* is the diffusion coefficient. This definition implies that $\tau_{F}$ (also indicative of the overall duration of pre-Fickian subdiffusion) increases as the inverse diffusion coefficient, on approaching the glass transition (i.e., on lowering temperature/increasing concentration).

**Figure 1:**
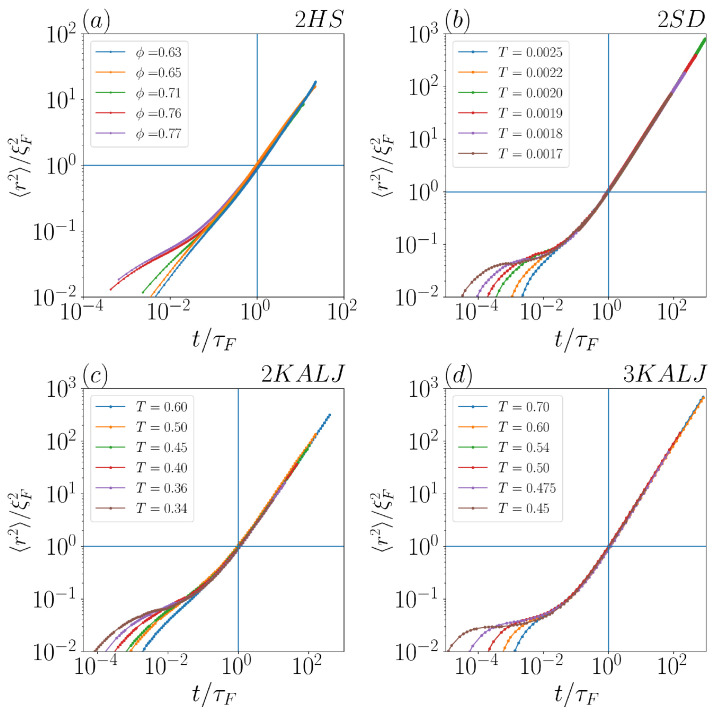
$\langle r^{2}\rangle /\xi_{F}^{2}$ as a function of $t/\tau_{F}$ in the four considered systems: (**a**) 2HS experiments at different volume fractions, and simulations at different temperatures of (**b**) 2SD, (**c**) 2KALJ and (**d**) 3KALJ (see Section 2 for the notation).

The above given definition is validated for each of the considered systems in the four panels of Figure 1, where $\langle r^{2}\left(t\right)\rangle$ is plotted for different temperatures/concentrations after shifting the abscissa and the ordinate by $\tau_{F}$ and $\xi_{F}$, respectively. The fact that, for each system, all MSD datasets show a linear time dependence starting from the point of coordinates (1,1) demonstrates how effective our identification of $\tau_{F}$ and $\xi_{F}$ is.

Concerning the upper time-boundary of FnGD, namely the time $\tau_{G}$ for the recovery of Gaussianity, for 2HS and 2DS, we follow the procedure of [30]. Precisely, $\tau_{G}$ is obtained by monitoring when the non-Gaussian parameter $\alpha_{2}\left(t\right)$ attains a properly low threshold, corresponding to the displacement distribution having in fact become indistinguishable from the Gaussian distribution of standard Brownian motion. It is worth noting that the temperature/area fraction dependence of $\tau_{G}$ is robust with respect to the threshold value, since $\alpha_{2}\left(t\right)$ data for different temperature/concentration collapse onto a unique master-curve [30] in the relevant long-time range. Thus, $\tau_{G}$ would only change by a constant factor on changing the threshold.

As a matter of fact, the so defined $\tau_{G}$ cannot be obtained for 3KALJ and 2KALJ, as $\alpha_{2}\left(t\right)$ data were not available in [29]. However, we will show in the following section that an alternative timescale $\tau_{x}$, yet closely related to $\tau_{G}$, will naturally arise from our analysis.

## 3. Results and Discussion

### 3.1. Displacement Distribution and Its Evolution in Two- and Three-Dimensions

**Figure 2:**
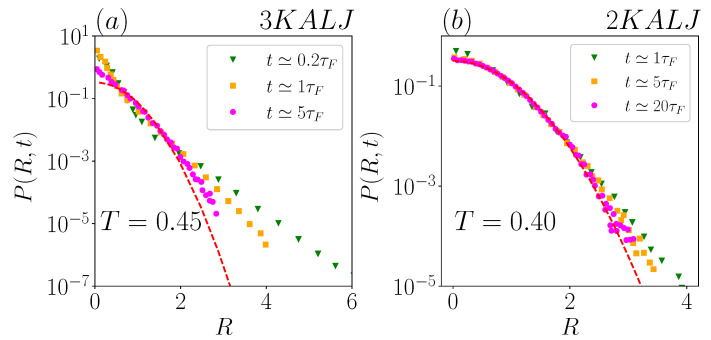
Rescaled distributions of particle displacements (see Section 3.1) at different times for the 3KALJ system at temperature T=0.45 (**a**) and for the 2KALJ system at T=0.40 (**b**). The red lines are the universal Gaussian distributions G(R).

Having identified in the previous section the time for the onset of Fickianity, $\tau_{F}$, we start here to analyze the displacement distribution functions p(r,t) from the data in Miotto et al. [29]. Notice that, at this stage, we will not consider deep pre-Fickian times, either in the ballistic or in the early sub-diffusive regime [44]. In Figure 2, we rescaled the data by defining $R=\frac{r}{\sqrt{\langle r^{2}\left(t\right)\rangle }}$, and $P\left(R,t\right)=p\left(r,t\right){\langle r^{2}\left(t\right)\rangle }^{d/2}$, where $\langle r^{2}\left(t\right)\rangle =\int r^{2}p\left(r,t\right)r^{d-1}dr$ is the MSD (We here have normalized p(r,t) as $\int_{0}^{\infty }p\left(r,t\right)r^{d-1}dr=1$). With this rescaling, the standard Brownian–Gaussian distribution $g\left(r,t\right)=e^{-\frac{dr^{2}}{2\langle r^{2}\left(t\right)\rangle }}\frac{d^{d/2}2^{1-d/2}}{{\langle r^{2}\left(t\right)\rangle }^{d/2}\Gamma \left(d/2\right)}$ becomes a time-independent curve: $G\left(R\right)=\frac{d^{d/2}2^{1-d/2}}{\Gamma (d/2)}e^{-dR^{2}/2}$. Hence, the adopted rescaling allows to clearly highlight deviations from Gaussianity and their time-evolution, and also to compare displacement distributions pertaining to different systems [4,33,35].

The distributions at temperature T=0.45 for 3KALJ and at T=0.40 for 2KALJ are included at various times in Figure 2a and b, respectively. Times are reported in units of $\tau_{F}\left(T\right)$.

The figure shows that, within the late sub-diffusive and early Fickian regime, the displacement distributions exhibit exponential tails $\propto e^{-R/\Lambda (t)}$ in both two- and three-dimensional systems, with a decrease of Λ(t) in time. We emphasize that, under the adopted representation, the presence of tails with different slopes in each panel unequivocally implies that the dimensional decay-length $\lambda \left(t\right)=\Lambda \left(t\right)\sqrt{\langle r^{2}\left(t\right)\rangle }$ does not scale as $t^{0.5}$, for both systems. This simple observation already questions the main conclusion presented in [29] on the 2KALJ system.

A noticeable difference between the two panels of Figure 2 is that all the distributions show clear exponential tails in the three-dimensional system, whereas, in the two-dimensional system, the displacement distribution at the longest available time, $t=20\tau_{F}$, seems to have reached the Gaussian limit, being in fact indistinguishable from the Gaussian mastercurve. This difference is simply due to the time-window spanned for the three-dimensional system being smaller, in terms of $t/\tau_{F}$, than for the two-dimensional system.

As a direct consequence of the already completed Gaussian recovery for the two-dimensional system at $t=20\tau_{F}$, it is apparent that any exponential fit to the tails of P(R,t) for $t\geq 20\tau_{F}$ is definitely unreliable. All such fits in Ref. [29] should actually be considered as very local fits to what in fact are Gaussian distributions.

**Figure 3:**
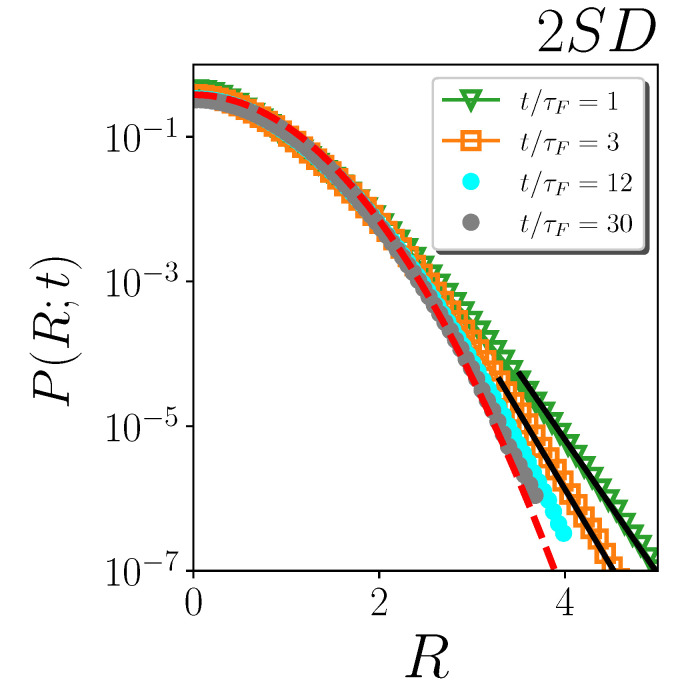
Rescaled distributions of particle displacements at different times in the non-dimensional plot, for 2DS at temperature T=0.0022. The red dashed line is the universal Gaussian distribution G(R). The black solid lines are exponential fits of the tails.

The ‘obvious’ existence of a limitation in time for the reliability of exponential fits to the tails is also evident in Figure 3, where we plot the distributions at various times and at a single temperature, as an example, for the numerical two-dimensional model investigated in Ref. [30]. At this temperature, exponential fits in Ref. [30] were performed only up to $t≲10\tau_{F}$, since they become inadequate at longer times, as Gaussianity is progressively recovered. This kind of precaution was, in fact, adopted in Ref. [30] for all the investigated temperatures.

**Figure 4:**
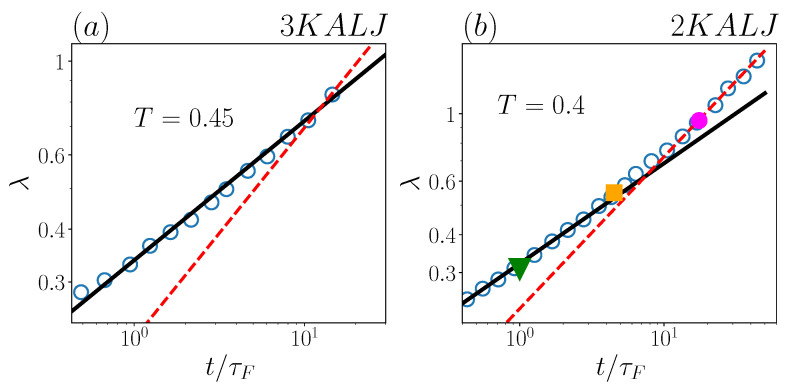
Decay length λ as a function of $t/\tau_{F}$ for (**a**) 3KALJ and (**b**) 2KALJ, for the indicated temperature. Black solid lines represent power-law $\propto t^{0.33}$, red dashed lines denote power-law $\propto t^{0.5}$. In panel (**b**), the three points with different color and marker-style correspond to the distributions of Figure 2b.

To quantitatively characterize the temporal evolution of the displacement distributions, we now draw our attention to the behaviour of the exponential decay-length λ(t). Firstly, we focus on the same two systems of Figure 2: the exponential decay length, as computed in [29], is reported in Figure 4, as a function of $t/\tau_{F}$. λ(t) has the same behaviour in both two-dimensional and three-dimensional systems (panel a and b, respectively) over around the first decade in $t/\tau_{F}$. In this range, including the early Fickian regime, the exponential tails of the displacement distributions are clear-cut, and the time-dependence of λ(t) is well captured by a $t^{0.33}$ power-law in both panels. We notice as an aside that this behaviour is already established in the late sub-diffusive regime ($t/\tau_{F}≲1$).

For the two-dimensional system (panel b), where data were available for quite a long time, λ(t) computed in [29] seemingly shows a crossover to a $t^{0.5}$ scaling for $t/\tau_{F}≳10$. In this time-range however, as previously noticed, exponential fits for this system are definitely not reliable, and therefore the corresponding λ values in Figure 4 must be disregarded. In other words, the “long-time regime” $\lambda \left(t\right)\propto t^{0.5}$ is an artifact, since no time-boundaries for the presence of exponential tails were considered in [29]. Incidentally, we notice that the lack of such time-boundaries was also evident in [29] on the short-time side, where λ values were attributed in the ballistic regime, even if the latter is known to be characterized by Gaussian displacement distributions [43].

**Figure 5:**
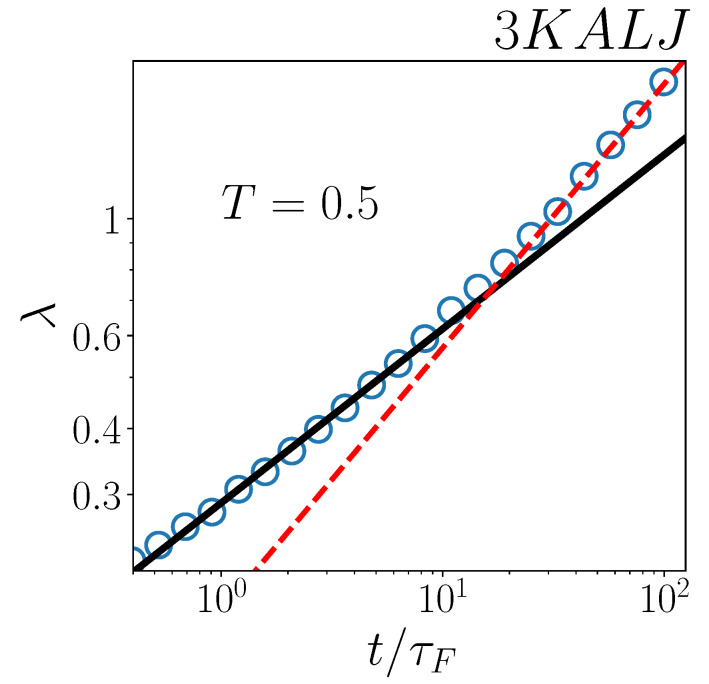
Decay length as a function of $t/\tau_{F}$ for the 3KALJ system at temperature T=0.5. Black solid line represents a power-law $\propto t^{0.33}$; red dashed line denotes a power-law $\propto t^{0.5}$.

At variance with the two-dimensional system (Figure 4b), three-dimensional simulations in Figure 4a, at T=0.45, are too short-lasting to verify whether any deviation from the $\lambda \left(t\right)\propto t^{0.33}$ behaviour emerges or not at long time periods.For this reason, in Figure 5, we examine λ(t), as computed in [29], for the three-dimensional system at a slightly higher temperature, T=0.5. It is interesting to note, in fact, that comparing the 3KALJ dynamics at T=0.5 with the 2KALJ at T=0.45 is particularly appropriate, since these two systems are at a similar “distance” from their respective Mode Coupling temperatures [43,45,46] and, therefore, similar dynamical features are expected. Now, at T=0.5, the simulated dynamics is long enough (in terms of $t/\tau_{F}$) to observe the same 0.33-to-0.5 power-law crossover in λ(t), as found in two-dimensions (Figure 4b), with the $t^{0.5}$ scaling stepping in charge at a similar time $t/\tau_{F}≃10$.

Also in the three-dimensional case, however, we argue that the long-time behaviour $\lambda \left(t\right)\propto t^{0.5}$ is an artifact, again arising from exponential fits having been performed in [29] within a (late) time-range, where Gaussianity of the displacement distributions is incipient. This inference is also supported by the independent computations in [47], showing that, at T=0.5 and $t=10\tau_{F}$, the non-Gaussian parameter of 3KALJ has in fact vanished.

**Figure 6:**
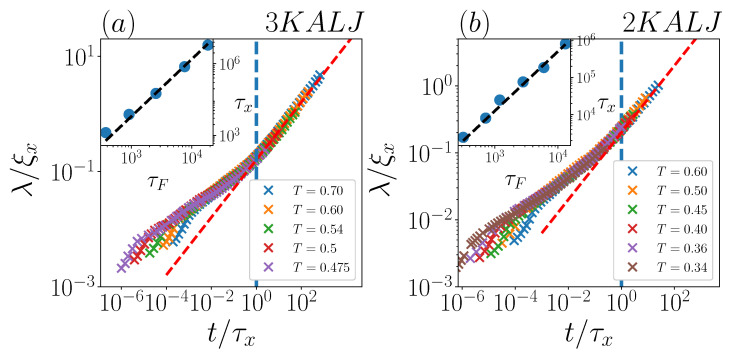
Non-dimensional decay length $\lambda /\xi_{x}$ as function of non-dimensional time $t/\tau_{x}$ for (**a**) 3KALJ and (**b**) 2KALJ and at the indicated temperatures. Red dashed lines represent power-laws $\propto t^{0.5}$, starting at $t/\tau_{x}≃1$ (vertical dashed lines). The insets in panels (**a**,**b**) show scatter plots of $\tau_{F}$ vs. $\tau_{x}$. In both cases, dashed lines are a power-law $\tau_{x}\propto \tau_{F}^{\gamma }$ with γ=1.6.

Inspecting the behaviour of λ(t) at different temperatures, as computed in [29], a similar scenario seems to emerge for both two- and three-dimensional systems (see Figure 6 below for plots including all considered temperatures): the only well-defined scaling for the decay length of the exponential tails is $\lambda \left(t\right)\propto t^{0.33}$; both the crossover and the (apparent) ensuing scaling law $\lambda \left(t\right)\propto t^{0.5}$ should be regarded as an indirect signal of Gaussianity restoring.

### 3.2. Master-Curves and Emerging Timescales

The upper time limit of the $\lambda \left(t\right)\propto t^{0.33}$ regime, in $t/\tau_{F}$ units, may in general depend on temperature. Indeed, in the emerging scenario, such time limit is controlled by the time for restoring of Gaussianity, $\tau_{G}$, whereas $\tau_{F}$ is a (lower) timescale related to onset of Fickianity: these two timescales do not have the same temperature dependence [30].

To address this issue, we re-analyze the λ(t) datasets in 3KALJ and 2KALJ at many different temperatures, reported in Ref. [29]. In this case, we also include short-time (very pre-Fickian) data. We rescale the abscissa of all λ curves by a shifting time $\tau_{x}$. For each dataset, $\tau_{x}$ is selected so as to make the apparent crossover occur at $t/\tau_{x}≃1$. Once the $\tau_{x}$ values are identified, the vertical axis for each λ curve is rescaled by the *diffusion length* $\xi_{x}=\sqrt{2dD\tau_{x}}$ associated with the corresponding $\tau_{x}$.

Using this rescaling, all data do collapse onto a single master-curve (apart from early-time deviations), as shown in Figure 6a,b for the two- and three-dimensional systems, respectively. These results clearly demonstrate that a common phenomenology, with a *unique* master-curve, arises for all systems, regardless not only of temperature, but also of space dimensionality, at odds with the main claim by Miotto et al. It is worth remarking that, by virtue of the $\tau_{x}$-based rescaling procedure, the previously described power-laws become more clearly visible, now covering several time decades. Notice that early-time deviations from the master-curve are essentially limited to the very pre-Fickian regime, especially to the ballistic range, where they take a “comb-like” shape. As discussed above, measurements of an exponential decay length in this regime are fully artificial. Similarly, the long-time $t^{0.5}$ behaviour, present both in two- and three-dimensional systems, comes from spurious late exponential fitting. The *real* scaling characterizing exponential tails, $\lambda \left(t\right)\propto t^{0.33}$, start in the sub-diffusive regime and persist in the early-to-intermediate Fickian one (i.e., within the true FnGD time-window).

In the emerging picture, the shifting time $\tau_{x}\left(T\right)$ is the characteristic timescale for the disappearing of exponential tails and the ensuing recovery of Gaussianity. We now explore the relation between $\tau_{x}$ and $\tau_{F}$ with varying temperature. Results are shown in the insets of Figure 6 for both two- and three-dimensional systems: we do find that data are fairly well described by a power-law relation $\tau_{x}\propto \tau_{F}^{\gamma^{\prime }}$, with $\gamma^{\prime }=1.6\pm 0.2$ for both systems. Interestingly, this exponent value is compatible with the exponent γ=1.8±0.2 for the power-law relation between $\tau_{G}$ and $\tau_{F}$ found for the two glass-forming liquids investigated in [30], thus hinting towards a close relationship between $\tau_{G}$ and $\tau_{x}$.

**Figure 7:**
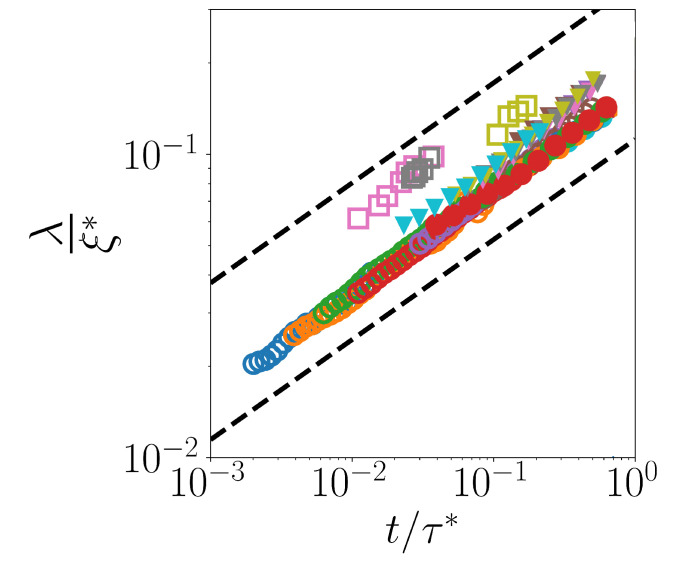
Non-dimensional decay length $\lambda /\xi^{*}$ as a function of non-dimensional time $t/\tau^{*}$ for the four considered systems within the Fickian regime $t>\tau_{F}$. The dashed lines represent power-law $\propto t^{0.33}$. Symbols as follows: squares for 2HS, empty circles for 2SD, triangles for 2KALJ and filled circles for 3KALJ.

Finally, in Figure 7, we report data for λ as a function of $t/\tau^{*}$, with $\tau^{*}$ being $\tau_{G}$ for the two two-dimensional systems considered in [30], and $\tau_{x}$ for the 2KALJ and 3KALJ considered in [29]. Correspondingly, λ has been rescaled by $\xi^{*}=\sqrt{2dD\tau^{*}}$. For all systems under examination, each dataset in the figure starts at $t=\tau_{F}$ and ends at $t=0.8\tau^{*}$, so as to remain in the regime where exponential fits are always reliable.

It is apparent from Figure 7 that all systems, in the considered time-window, display a common scaling $\lambda \left(t\right)\propto t^{1/3}$, independently from dimensionality, and from the specific form of the (isotropic) interaction potential. The figure also confirms that, for each system, data at different temperature (concentration) collapse onto a master curve, $\frac{\lambda (t)}{\xi^{*}}=C{\left(\frac{t}{\tau^{*}}\right)}^{1/3}$, with *C* independent from temperature (concentration).

## 4. Conclusions

The results illustrated in this paper point toward a strong similarity in the long-time (Fickian yet non-Gaussian) dynamics of two- and three-dimensional glass-forming liquids. Drawing on data from four different glass-formers, our analysis, in fact, suggests that the behaviour of the tails of the displacement distribution function is universal near the glass transition, at least for systems with isotropic interactions. Of course, other aspects of glassy dynamics, e.g., the caged particle motion, may show differences between two and three dimensions, as indicated by a body of recent works, including Refs. [48,49,50,51,52]. Overall, universal and non-universal behaviours may coexist close to the glass transition, possibly emerging over different time- and length-scales.

Interesting perspectives include, first of all, the validation of the present findings for other glass-forming systems, and their possible confirmation (or variation) towards deeply supercooled conditions, i.e., very close to glass transition. A further interesting issue concerns the time-range preceding FnGD, namely the sub-diffusive regime, with particular emphasis on the quest for FnGD precursors. Finally, the insights here obtained for glass-forming liquids may likely help rationalizing the emergence of FnGD in other systems up to macroscopic scales, including applicative contexts, 4d e.g., adhesion, lubrication and sorption [53,54,55].

## Data Availability

The data that support the findings of this study are available from the corresponding author upon reasonable request.

## Abbreviations

The following abbreviations are used in this manuscript:

FnGD	Fickian non-Gaussian Diffusion
MSD	Mean Square Displacement
3KALJ	Three-dimensional Kob and Andersen Lennard–Jones system
2KALJ	Two-dimensional Kob and Andersen Lennard–Jones system
2HS	Two-dimensional hard-sphere system
2SD	Two-dimensional soft disk system
MD	Molecular Dynamics
